# COVID-19-Induced Cavitary Lesion: A Rare Presentation

**DOI:** 10.7759/cureus.18723

**Published:** 2021-10-12

**Authors:** Steven Douedi, Sydney Kauffman, Mohammed AlAzzawi, Swapnil V Patel, Ahmad Abu

**Affiliations:** 1 Internal Medicine, Jersey Shore University Medical Center, Neptune, USA; 2 Internal Medicine, St. George's University School of Medicine, True Blue, GRD; 3 Pulmonary and Critical Care Medicine, Jersey Shore University Medical Center, Neptune, USA

**Keywords:** severe acute respiratory syndrome coronavirus 2, hemoptysis, cavitary, pneumonia, covid-19

## Abstract

The coronavirus disease 2019 (COVID-19) pandemic has resulted in widespread infection with significant morbidity and mortality. Primarily involving the respiratory system, COVID-19 has also been known to cause systemic findings. Cavitary lesions in the setting of COVID-19 have been rarely reported in literature and the treatment and management of these lesions are poorly understood and defined. We present a case of a patient with a history of COVID-19 infection found to have cavitary lesions, eventually improving with supportive care. While our patient showed marked improvement with observation, more extensive studies and patient populations are needed to guide clinicians and cavitary lesion management in the setting of COVID-19.

## Introduction

Coronavirus disease (COVID-19) has been recognized as a devastating pandemic leading to sequelae of various organ system involvement. Until now, COVID-19 has infected over 195 million individuals and led to over 4 million deaths globally [1]. Identified as an enveloped, non-segmented positive-sense RNA virus belonging to the beta-coronaviridae family, various pharmaceutical companies have developed an mRNA-based vaccine of which over 3.8 billion individuals have received according to the World Health Organization [1,2]. Known to primarily involve the respiratory system, several meta-analyses have reported common findings on chest computed tomography scans such as peripheral ground-glass opacities, air bronchograms, linear opacities, consolidation, and in late stages traction bronchiectasis [3,4]. Rarely, reports and literature have described an association between COVID-19 infection and the development of cavitary lesions [5,6]. We report a case of an otherwise healthy male with a history of COVID-19 infection presenting with hemoptysis and ultimately found to have multiple cavitary lesions associated with COVID-19.

## Case presentation

**Figure 1:**
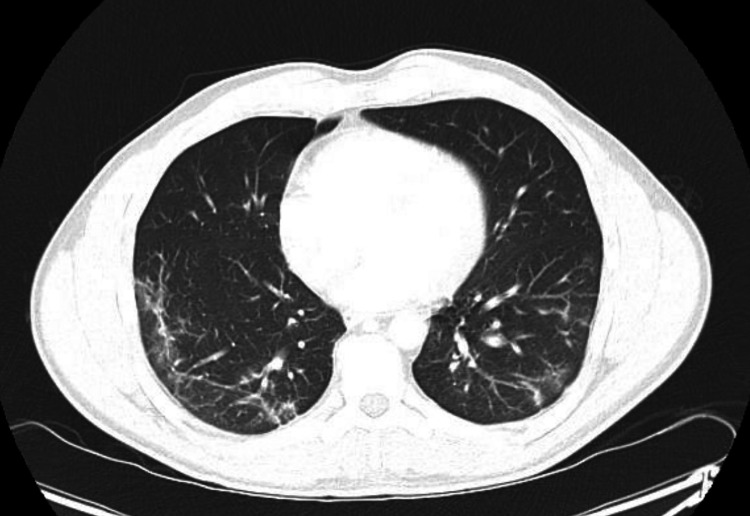
CTA of the chest. Computed tomography angiography (CTA) scan of the chest showing multifocal patchy infiltrates with slight mosaic pattern but no noted cavitary lesion.

A 43-year-old male with a medical history of Hashimoto's thyroiditis and COVID-19 infection diagnosed by reverse transcription polymerase chain reaction (RT-PCR) four months prior to admission presented to the office with complaints of hemoptysis. During his COVID-19 infection, his primary symptoms were a non-productive cough without shortness of breath or hypoxia, for which he was treated supportively. He denied any other complaints; including fevers, chills, shortness of breath, hematuria, occult blood in his stool, weight loss, or night sweats. He also denied any recent travel or sick contacts. Computed tomography (CT) angiography of the chest obtained during his initial infection four months prior showed multifocal patchy infiltrates with a slight mosaic pattern but no noted cavitary lesion (Figure 1).

**Figure 2:**
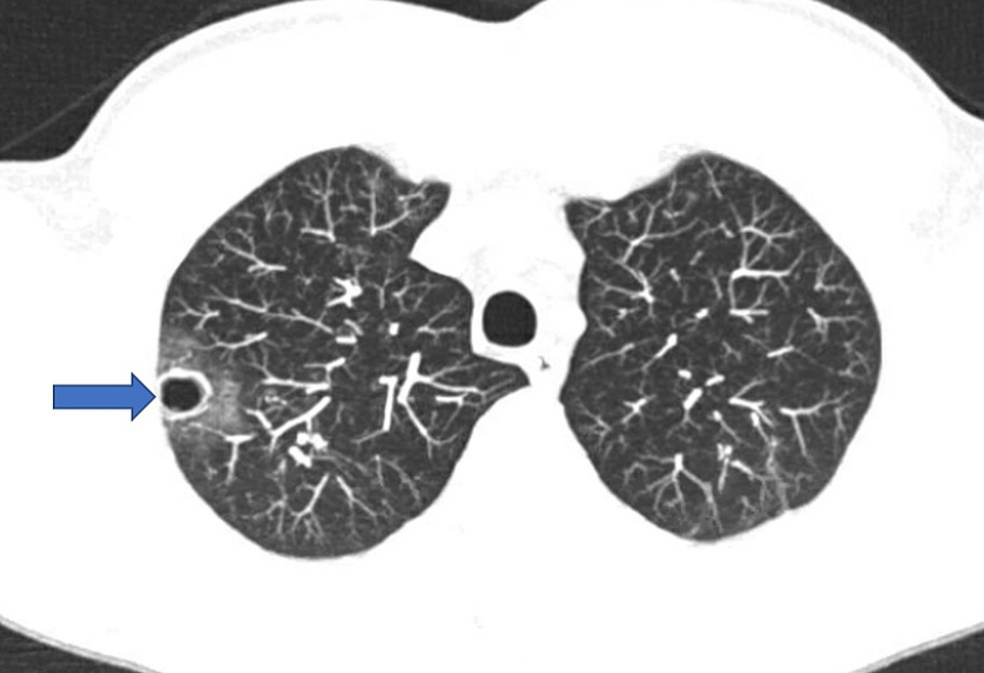
CT scan of the chest. CT scan of the chest showing scattered reticular, ground-glass, atelectatic and fibrotic changes again seen in both lungs. These are slightly worsened compared to Figure 1 especially in the right upper lobe where there is a groundglass patchy infiltrate of 5 cm in size with associated new cavity of 2 cm in the right middle lobe (blue arrow).

He remained hemodynamically stable and was advised to follow up with a pulmonologist at that time however was lost to follow up. Six months after his initial presentation, he presented to the outpatient office again with complaints of hemoptysis without any associated symptoms. A repeat CT scan of the chest was obtained showing multiple cavities in the right upper and middle lobe (Figure 2).

**Figure 3:**
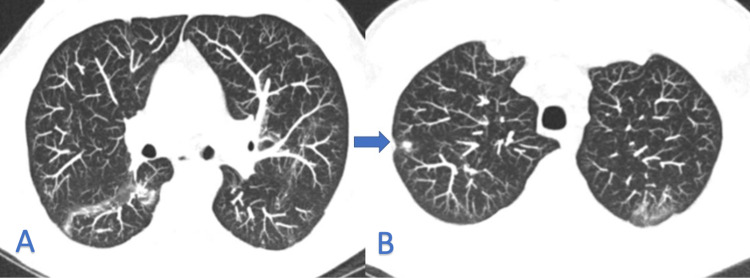
CT scan of the chest on follow-up. CT chest showing persistent however slightly improved patchy ground-glass opacities seen throughout the lower lobes (panel A and B). The ground-glass upper lobe opacities have improved however there is a persistent 4 mm right upper lobe nodule (blue arrow in panel B).

Laboratory findings at this visit including complete blood count, complete metabolic panel, antinuclear antibody, anti-neutrophil cytoplasmic antibody, and QuantiFERON were within normal limits. He was evaluated by the pulmonary team and a bronchoscopy was performed. Bacterial, acid-fast, and fungal cultures were obtained and showed no growth. Bronchoalveolar lavage and cytology showed no abnormalities. He was advised to continue close follow-ups and, one month later, the patient's hemoptysis had resolved. A treatment plan was made to perform routine serial CT scans to monitor for growth or progression of cavitary lesions. Two months after his initial presentation, a repeat CT scan of the chest showed resolving ground-glass opacities and significant improvement of nodules (Figure 3). 

## Discussion

Chest computed tomography scan has been found to be the most helpful for diagnosis, management, and follow-up of COVID-19 due to its high sensitivity at nearly 97% [7]. Thus far, the most common findings of COVID-19 on chest CT are ground-glass opacities, vessel enlargement, consolidation, air bronchograms, and bronchiectasis found in late stages of disease [8]. Our literature search found that cavitations were a very uncommon finding on chest CT in the setting of COVID-19 infections [5,6]. More specifically, many studies reported a temporal change in chest CT findings in follow-up examinations, including a transition from isolated ground glass opacities to a superimposition of ground-glass opacities with cavitation over time [9]. 

Another study looking at 104 symptomatic patients with COVID-19 from seven isolation centers was retrospectively analyzed in early 2020 and showed that 1% of their patients showed cavitations on chest CT imaging [10]. Hugo et al. discovered that 28 studies comprising 3,466 patients showed 0.7% of patients had cavitation found on chest CT [11]. The patient presented in our case did not have the typical findings of COVID-19 on imaging defined in the literature. The cavitary lesions seen in our patient were thoroughly worked up to find the underlying etiology, consistent with the more common causes of cavitary lesions such as mycobacterium, infectious, parasitic, or autoimmune [5]. Given the extensive yet unremarkable work-up for the cavitary lesion etiology, COVID-19 appeared to be the most probable cause. 

The pathophysiology of cavitary lesions in the lung has been well described in the literature. In other disease processes, these lesions are suspected to be primarily due to underlying various types of necrosis or malignancy [12]. In post-mortem analysis of COVID-19-infected patients with cavitary lesions, diffuse alveolar damage or intra-alveolar hemorrhage and necrosis have been implied as possible causes. However, this is still unknown and poorly understood [5,13]. 

## Conclusions

Although this finding is rare, it is still important to understand the implications of this finding on chest CT and define a possible course of management. As of now, these cavitary lesions have been managed conservatively. Further studies are needed, and patients with cavitary lesions due to COVID-19 infection should be closely monitored. This case will hopefully contribute to the constantly growing and changing knowledge we have about the COVID-19 virus and its consequences in our patients.
